# Carbon Nanotube Electron Emitter for X-ray Imaging

**DOI:** 10.3390/ma5112353

**Published:** 2012-11-16

**Authors:** Je Hwang Ryu, Jung Su Kang, Kyu Chang Park

**Affiliations:** Department of Information Display and Advanced Display Research Center, Kyung Hee University, Seoul 130-701, Korea; E-Mail: jyoo@khu.ac.kr (J.H.R.); kangjungsu@khu.ac.kr (J.S.K)

**Keywords:** carbon nanotube, X-ray, resist assisted patterning

## Abstract

The carbon nanotube field emitter array was grown on silicon substrate through a resist-assisted patterning (RAP) process. The shape of the carbon nanotube array is elliptical with 2.0 × 0.5 mm^2^ for an isotropic focal spot size at anode target. The field emission properties with triode electrodes show a gate turn-on field of 3 V/µm at an anode emission current of 0.1 mA. The author demonstrated the X-ray source with triode electrode structure utilizing the carbon nanotube emitter, and the transmitted X-ray image was of high resolution.

## 1. Introduction

Carbon nanotubes (CNT) show excellent properties that make them attractive for several nanoelectronic device applications, including electron emitters [1]. CNTs exhibit characteristics, such as high aspect ratio, high thermal conductivity, and low chemical reactivity, which also make them suitable for the functionalized fabrication of X-ray tubes, and X-ray fluorescence (XRF), X-ray diffraction (XRD) [2,3]. Stable field emission is the key requirement for CNTs in high performance electron emission source applications [4]. Thus, there is a critical need for novel methods for growing CNTs. Electron sources for X-ray generation in tomosynthesis require an anode current that is greater than 100 mA and endures high anode voltage [5,6].

Conventional CNT emitters such as electrophoresis process, chemical vapor deposition, and printing method process, do not meet this requirement because they quickly degrade during high current operation. Also, emission current is not enough for those devices. Stable and reliable electron emission is also necessary for extended operation. For enhanced and stabilized electron emission, electron sources must strongly adhere to the substrate, be robust under environmental stress, and possess sharp tips, which do not change and degrade during operation. 

In this paper, we describe the growth and post-treatments of CNTs for making high-performance emitters. The CNT emitter grown using the RAP process can prevent chemical reactions of carbon with gases in the environment and improve the stability of the CNTs. Also, by using the CNT emitter, we demonstrated the proto-type X-ray imaging system based on CNT emitter. The structural and electron emission properties of the emitters and their application of X-ray imaging are discussed.

## 2. Experimental Section

### 2.1. Growth of Carbon Nanotube for High-Performance Emitters

**Figure 1:**
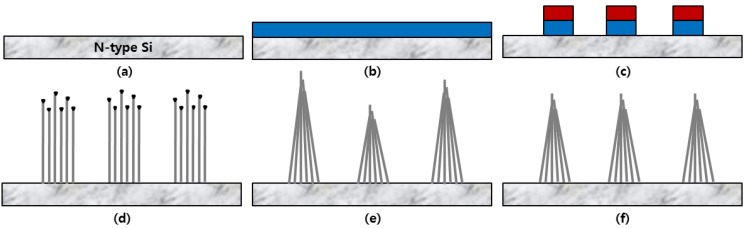
Process flow of carbon nanotubes (CNT) emitters for X-ray source. (**a**) Cleaned Si substrate; (**b**) Ni catalyst deposition; (**c**) Patterning and forming for seed formation; (**d**) CNT growth; (**e**) hydrofluoric (HF) treatment; (**f**) Electrical aging treatment.

The CNTs were grown using a resist-assisted patterning (RAP) process, which has been reported in detail elsewhere [7]. For the RAP process, silicon substrates were used to grow the CNTs in Figure 1a. As shown the Figure 1b, the nickel catalyst layer with thickness of 10 nm was deposited on the silicon substrate using radio frequency magnetron sputtering. The nickel catalyst layer was patterned using photo-resist. The resist was patterned elliptical shape of 2.0 × 0.5 mm, and inner pattern is 3 µm sized islands with an island-to-island pitch of 15 µm. The 15 µm pitch is designed to reduce field screening by nearest emitters. The resist was not removed for seed formation and is required for CNT growth. The unwanted nickel catalyst was etched, and then the substrate was annealed in a high-temperature furnace in Figure 1c. The annealing temperature and time in this study were 600 °C and 30 min, respectively. After annealing, the nickel catalyst separated into small grains for the growth of the CNTs. CNTs can grow where there is carbon-network formation after the annealing process. In Figure 1d, the CNTs were grown in a triode direct current-plasma enhanced chemical vapor deposition dc-PECVD system with a mesh grid placed 10 mm above the substrate holder electrode. With this triode dc-PECVD system, the growth of CNTs is easily enhanced and inhibited by strong positive and negative bias voltages on the mesh electrode, respectively [8].

The CNT density and length can be changed easily by changing the mesh electrode bias and polarity. The mesh electrode bias voltage can alter the CNT growth mechanism by modifying the flux of the ions impinging on the substrate. A positive mesh bias increases the ion flux and electric field, resulting in increased CNT length and density the higher the bias, the higher the length and density. However, a negative mesh bias, resulting in a reduced ion flux, reduces CNT density and growth rate probably because of catalyst particle poisoning by an amorphous carbon deposit that cannot be removed through sputtering.

After growing the patterned CNT emitters of elliptical shape in Figure 1d, they were performed the post-treatment with hydrofluoric (HF) acid. The interface between the CNTs and substrate was robust enough to withstand HF treatment. After HF treatment, CNT emitters were get-together in the tip position, resulting cone-shape formation. The cone shape formation could enhance stability of electron emission current. The HF treatment is a very simple and cost-effective process for improving the electronic properties of CNTs emitter [9].

And then, we carried the 2nd post treatment which the effect of electrical aging in high vacuum chamber. By the electrical aging treatment, the nickel catalysts were removed from top of nanotube, and the electron emission current was enhanced and the emission stability was improved for high-performance X-ray source. Finally, we can obtain the sharpened and uniform emitters [10].

The structural properties of the CNTs were characterized through field emission scanning electron microscopy (FE-SEM) and transmission electron microscopy (TEM).

### 2.2. X-ray Generation with the High-Performance CNT-Emitters

**Figure 2:**
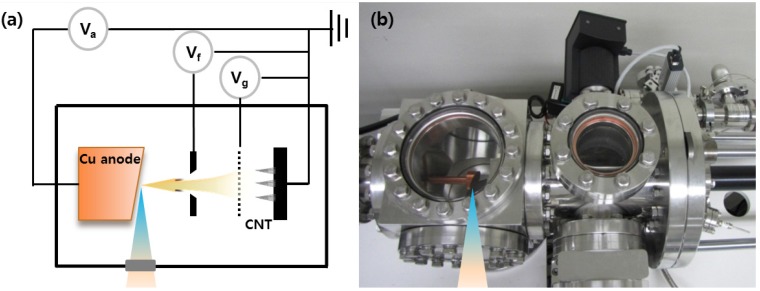
(**a**) Schematic diagram and (**b**) a photo image of the X-ray generator with CNT emitter.

The X-rays were generated with a four electrodes X-ray system including electrodes with anode, focusing, gate, and cathode. The focal spot size of X-ray generation was found to depend on the emitter arrays geometry. Figure 2a shows the schematic diagram of the X-ray generation system. The electrons were extracted from grounded CNT emitters by controlling the gate electrode, and became focused electron with adjusting focus voltage. The focused electrons were rapidly decelerated by the anode target to produce characteristic X-rays. The distance between anode with a molybdenum and gate was maintained at 8 mm. The base pressure of the X-ray generation system was kept at 1 × 10^−7^ Torr. 

Figure 2b shows the photograph image of triode type X-ray system. In these experiments, we use vacuum system for X-ray generation instead of vacuum sealing tube. The vacuum sealing technique for colt cathode is not matured. The full size of the system 20 cm × 20 cm × 50 cm (H × V × W) and sample was loaded through load-lock chamber and X-ray generation chamber keep ultra-high pressure. The CNT electron source was delivered through home-made delivery systems. The X-ray system design utilizing CNT cathode may be a significant advance in X-ray technology development and could lead to portable and miniature X-ray sources for medical and industrial applications.

## 3. Results

### 3.1. CNT Emitters for High-Performance X-ray

**Figure 3:**
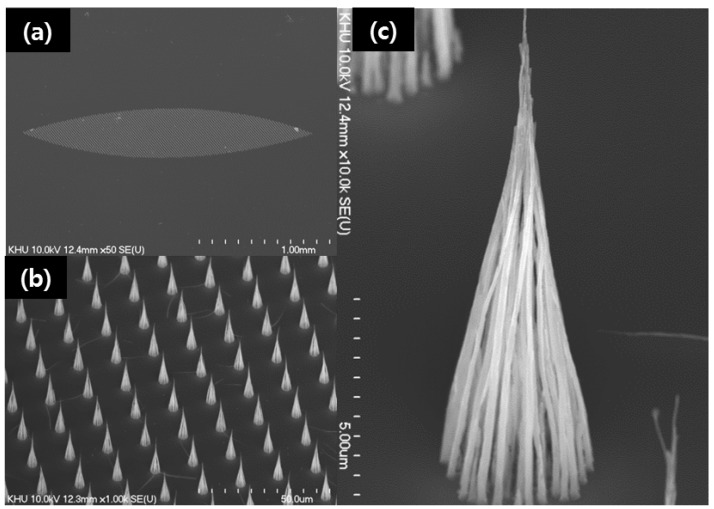
SEM images of the (**a**) elliptical emitter with 2.0 mm × 0.5 mm formed by RAP process; (**b**) magnified image of CNT array emitters with 3 µm islands and 15 µm pitch; (**c**) a magnified image of one CNT-bundles.

For the isotropic focal spot at anode target, the emitter is an elliptical area of 2.0 mm × 0.5 mm as shown in Figure 3a [11]. The initial CNTs without post-treatment are vertically aligned and formed several nickel catalysts on top of multiwall CNTs [7]. However, after post-treatments, there is no nickel catalyst. A SEM image of the vertically aligned CNTs is shown in Figure 3b. One CNTs bundle had a diameter of 3 µm, and they were precisely pitched every 15 µm. The island size and pitch of the CNTs could be controlled through the catalyst fabrication process as shown in Figure 1c. The magnified image of CNTs is shown in Figure 3c. 

The figure clearly shows that the CNT had a sharp tip and a wide bottom. It was vertically aligned and well positioned in the predefined island area. The length of the CNT was over 10 µm. The one CNT-bundle emitter was cone shaped. The tip apex measured a few nanometers in diameter, and the diameter of the bottom region in contact with the substrate was equal to the pre-patterned dot area of the 3 µm island. The adhesion of conventional single CNTs onto the substrate is weak [12]. This is thought to increase the adhesion between the CNTs and the substrate, also to reduce the screening effect between emitters and adjacent emitter [13].

**Figure 4:**
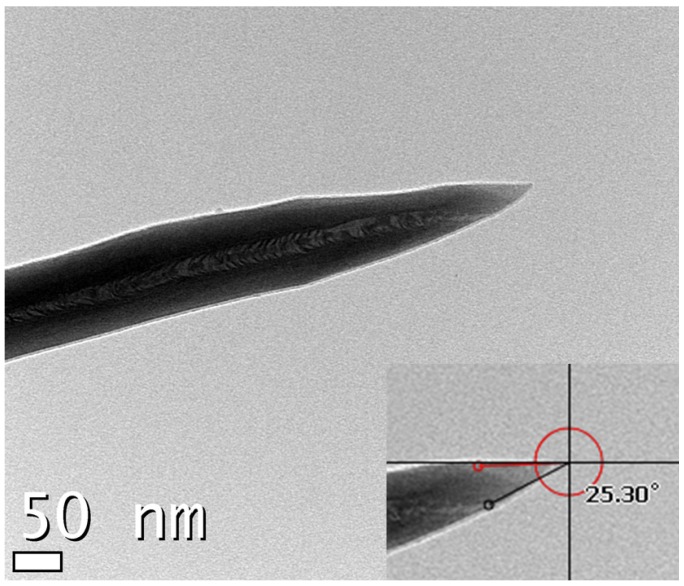
TEM image of a magnified image of the tip apex of a CNT emitter.

As shown in Figure 4, the diameter and angle of the tip were in the nanometer range and around 25.30°, respectively. The emitter tip was cone shaped with a sharp tip. The tip apex measured a few nanometers in diameter. There was one CNT-bundle emitter with multiple rods in the bottom. To understand the structural properties, Additional details concerning the mechanism can be found in a previously published report [12].

### 3.2. X-ray Generation with the High-Performance CNT-Emitters

**Figure 5:**
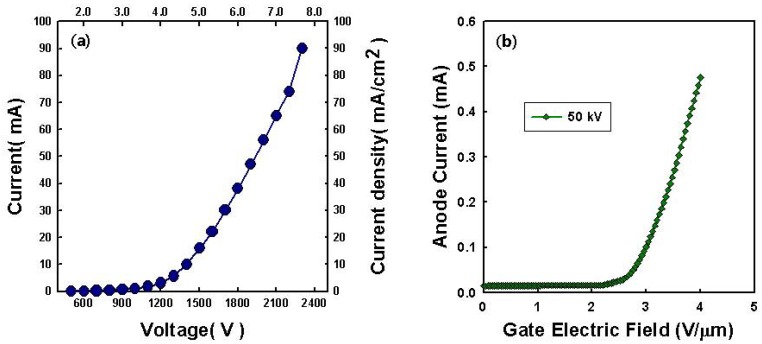
Field emission properties of (**a**) diode and (**b**) triode measurement for X-ray imaging.

Figure 5 shows the electron field emission properties of the elliptical CNT emitters. Prior to obtaining X-ray imaging, we performed the field emission measurements on the diode structure. Through the diode structure, we can establish the material field emission properties without module problems, such as anode melting and arcing effect between gate and cathode. The obtained emission current on direct current mode is 90 mA at 2100 V (7.5 V/µm). For X-ray imaging, the emission current was measured using a four electrodes structure as shown in Figure 2. The acceleration voltage was applied to anode of 50 kV. The cathode current of 0.5 mA was obtained at a gate electric field of 4 V/µm. The anode current was measured by adjusting the gate electrode in the dc mode. The transmission rate to anode target was ~70% from elliptical emitter.

**Figure 6:**
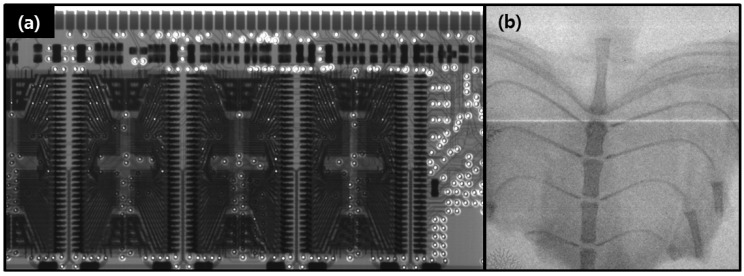
X-ray images of (**a**) dynamic random-access memory; (**b**) chest region of mouse.

Figure 6 shows X-ray images of dynamic random-access memory and the biological object. The electrons were extracted from the emitter by the positive gate field 4 V/µm applied to the gate electrode. For X-ray generation, the anode voltage and current was maintained at 50 kV and 0.5 mA, respectively, with an exposure time of 3 s. We can clearly see the transmitted-ray images of various objects. Based on this research, in future we will apply more emphasis to X-ray properties of our CNT electron based X-ray generation.

## 4. Conclusions

A CNT based field emission X-ray source with four electrodes including anode, focusing, gate, and cathode has been demonstrated. The high-performance CNT emitter grown using the RAP process appears to be an excellent candidate for electron sources for X-ray generation. The CNT-bundles emitters of elliptical shape were fabricated using the RAP with lithography process. In the post-treatment process with HF dipping and electrical aging, we could then modify emitters to align the cone shape. The cone shape emitters produced an enhanced and stable electron emission. This enhanced emission combined with the ability to control emitter pitch, produces an enhancement of the X-ray image. With these emitters, we obtained high resolution X-ray images of on industrial product and a small animal.
